# The Influence of Human-Milk Substitutes Marketing on Breastfeeding Intention and Practice among Native and Immigrant Brazilians

**DOI:** 10.1177/08903344221104717

**Published:** 2022-07-06

**Authors:** Cosima Lisi, Henrique Barros, Alexandre Faisal-Cury, Alicia Matijasevich, Cláudia de Freitas

**Affiliations:** 1 EPIUnit - Instituto de Saúde Pública, Universidade do Porto, Porto, Portugal; 2 Laboratório para a Investigação Integrativa e Translacional em Saúde Populacional (ITR), Porto, Portugal; 3 Departamento de Medicina Preventiva, Faculdade de Medicina FMUSP da Universidade de São Paulo, Sao Paulo, Brazil

**Keywords:** Brazil, breastfeeding, cultural norms, ethics, formula marketing, Human Migration, International Code of Marketing of Breast Milk Substitutes, Portugal, qualitative methods

## Abstract

**Background:**

The International Code of Marketing of Breast-Milk Substitutes is a global public health policy aiming to protect breastfeeding from the influence of human-milk substitutes marketing. Brazil is one of the few countries substantially implementing it. Most countries adopted selected provisions, including Portugal.

**Research Aim:**

To explore whether Brazilians’ perspectives about breastfeeding intention and practice are influenced by human-milk substitutes marketing upon migration to Portugal.

**Methods:**

A qualitative, prospective, cross-sectional survey design was conducted in Brazil and Portugal (2018–2019). Qualitative semi-structured interviews were performed with native (*n* = 16) and immigrant (*n* = 15) Brazilians. Women aged 18 or above, mothers of 0–12 month infants, and without contraindications to breastfeed, were eligible for the study. Heterogeneity sampling was employed based on socioeconomic status and infants’ age. Content analysis was conducted using NVivo.

**Results:**

Brazilian immigrants were more aware of the potential negative influence of human-milk substitutes marketing than natives. Sociocultural factors contributed to Brazilian immigrants being less permeable to the influence of human-milk substitutes marketing in the host country, where a less protective breastfeeding environment was perceived.

**Conclusions:**

Sociocultural factors including breastfeeding promotion strategies and a strong breastfeeding culture in the home country appear to play a protective role on breastfeeding intention and practice among Brazilians migrating to Portugal.

## Background

Human milk is the ideal food for the healthy growth and development of infants (Victora et al., 2016). The World Health Organization (WHO, 2021) recommends early initiation of breastfeeding within 1 hr of birth, exclusive breastfeeding up to 6 months of age and continued breastfeeding up to 2 years of age or beyond. However, only 44% of infants aged 0–6 months are exclusively breastfed globally (WHO, 2021). Breastfeeding practices are influenced by individual (maternal and infant characteristics and relationship) and structural factors (sociocultural and market context) (Rollins et al., 2016). The study of structural market factors has been gaining prominence, however, given its increasing role in shaping breastfeeding intention and practice (Baker et al., 2021; Harris & Pomeranz, 2020; Hastings et al., 2020). Exposure to human-milk substitutes (HMS) marketing can instill doubt about optimal infant feeding, reducing mothers’ confidence in their ability to breastfeed and significantly increasing breastfeeding cessation (Baker et al., 2020; Harris & Pomeranz, 2020; Lisi et al., 2021b)

In 1981, the World Health Assembly approved the International Code of Marketing of Breast-milk Substitutes (IC), a global public health policy aiming to regulate unethical and inappropriate marketing of HMS (WHO, 1981, 2017). It is the responsibility of each member state to adopt national legislation, or other suitable measures, to bring the IC provisions into effect (WHO, 1981).

In 2020, only 25 countries had substantially implemented the IC (WHO, United Nations Children’s Fund [UNICEF] & the International Baby Food Action Network [IBFAN], 2020). Brazil is one of those countries in South America (Law No. 11.265, 2006) and it has been acknowledged worldwide as an example in breastfeeding promotion (Rollins et al., 2016). The IC was adopted by the Brazilian Health Council (1988) as a resolution within the National Breastfeeding Promotion Program in 1988. Subsequent revisions of this resolution resulted in the so called “Brazilian Norm for Commercialization of Food for Nursing and Children of First Infancy, Rubber Nipples, Pacifiers, and Nursing Bottles” (NBCAL), which was transposed into law in 2006 (Rodrigues et al., 2021). This law prohibits the promotion of the following products: Infant formulas, follow-up formulas, nourishing formulas presented and/or indicated for high-risk newborns, rubber nipples, nursing bottles, and pacifiers. The promotion of toddler milks and other types of milk under the scope of the law is allowed, provided that the recommendation of breastfeeding up to 2 years or beyond is given. The law regulates the marketing of all the products under the scope of the IC, thus positioning Brazil among the countries with the highest degree of IC implementation (WHO, UNICEF & IBFAN, 2020). Portugal is, conversely, one of the European countries where only a few IC provisions were adopted (WHO, UNICEF & IBFAN, 2020). In 2020, the European Union enforced Regulation 2016/127 (2016), which restricts promotional and commercial practices of infant formula. The Portuguese government has adopted a few provisions of the IC through the Decree Law No. 62, 2017, which also prohibits the promotion of infant formula (Decree Law No. 62, 2017).

While a favorable sociocultural context may work to protect breastfeeding, poor IC implementation may create a less enabling environment for breastfeeding (Pérez-Escamilla, 2020). However, less is known about how migration to countries with limited legal provisions of the IC influences women’s perspectives about infant feeding practices. Researchers have shown that immigrant status is an important determinant of breastfeeding (Dennis et al., 2019). Immigrant women have better breastfeeding outcomes than their native counterparts (Dennis et al., 2019) and that also has been observed in Portugal (Kana et al., 2018). Nevertheless, HMS marketing has been associated with breastfeeding discontinuation among both Portuguese native and immigrant mothers (Lisi et al., 2021b). Brazilians are the largest migrant group in Portugal, making up 22% of the total migrant population. As a former Portuguese colony, Brazil shares historical and linguistic ties with Portugal, and they have the same official language. Pursuing academic studies has taken over family reunification as the leading reason for migration to Portugal (Oliveira, 2020). Brazilians have the highest proportion of women (59.4%) from all migrant groups and are one of the groups that has contributed the most to the total live births (3.1%) in Portugal during 2018 (Oliveira, 2020).

**Key Messages**

- Little is known about how migration between countries with differing implementation of the International Code of Marketing of Breast-Milk Substitutes might influence breastfeeding intention and practice.
- Brazilian immigrants who migrated to Portugal reported more contact with human-milk substitutes marketing than Brazilian natives, but that did not change their breastfeeding intention and practice.
- A strong breastfeeding culture and continuous breastfeeding promotion strategies in the home country seem to play a protective role against the negative influence of human-milk substitutes marketing among Brazilians living in Portugal.

Based on this evidence, it is important to inquire whether Brazilian immigrants living in Portugal, where the IC is not fully implemented, are exposed to market factors and if that leads to suboptimal infant feeding. Thus, we conducted a qualitative study to explore whether Brazilians’ perspectives about breastfeeding intention and practice are influenced by HMS marketing upon migration to Portugal.

## Methods

### Research Design

A qualitative, prospective, cross-sectional survey design was conducted in two locations (DiCicco-Bloom & Crabtree, 2006). This design enabled a comparison between participants’ perspectives, by conducting qualitative semi-structured interviews in Portugal and Brazil, where the IC is implemented to distinct degrees. This study received ethical approval from the Ethics Committee of the Institute of Public Health of the University of Porto (Proc. No. CE18085/May 10, 2018) in Portugal and the Committee for Ethics in Research of the University of São Paulo (Proc. No. 3.241.864/April 3, 2019) in Brazil.

### Setting and Relevant Context

Brazil is the largest country in South America and has 26 federal states distributed through five major geographic regions. The state of São Paulo, where the study was conducted, is highly industrialized, representing 28.9% of Brazil’s industrial GDP (Confederação Nacional da Indústria, 2021). It belongs to the Southeast region where the exclusive breastfeeding rate under 6 months is at 49.1%, similarly to the national rate (45.8%; Federal University of Rio de Janeiro, 2021). Differences in exclusive breastfeeding rates were observed among the five Brazilian regions but they were not statistically significant.

Portugal is a European high income country where progress in the BFHI implementation and maternity leave has led to 31.9% of children being exclusively breastfed up to 6 months in 2012–2014 (Kislaya et al., 2020). Exclusive breastfeeding duration is higher among immigrants living in Portugal, when compared to Portuguese natives (Lisi et al., 2021b). Similar results were found in a population-based study conducted in the Porto Metropolitan Area (Kana et al., 2018) in the North region of Portugal, which represents 30.2% of Portuguese GDP (Pordata, 2021), and where we conducted our qualitative interviews. Nevertheless, exposure to free samples and discounts of HMS has been associated with decreased breastfeeding rates among both natives and immigrants (Lisi et al., 2021b).

### Sample

Native Brazilians living in the state of São Paulo and immigrant Brazilians living in Portugal were eligible for this study. Inclusion criteria were: (a) being at least 18 years of age and (b) having a child between 0–12 months. We excluded women with contraindications to breastfeeding. Purposive heterogeneity sampling was employed to guarantee maximum variation of perspectives based on two criteria: Women’s socioeconomic status (i.e., maternal education and household income) and infants’ age, which was deliberately chosen to reduce recall bias. All eligible women were invited to participate in this study by the first author. No monetary compensation was given to participants. Interviewing continued until thematic saturation was reached (Guest et al., 1995). This methodological principle was used to determine the size of our sample. A total of 16 Brazilian natives and 15 Brazilian immigrants participated in the study. Data saturation was reached during analysis, which attests to the sample size being adequate to respond to our research question.

### Data Collection

Between June 2018 and January 2019 individual, in-person, semi-structured interviews were conducted with Brazilian immigrants, and between May–June 2019 with Brazilian natives. In Portugal, participants were recruited through the project *Migrants and perinatal health: Barriers, incentives and outcomes* (baMBINO)—a nationwide study that investigated perinatal health and the healthcare experiences of immigrants living in Portugal and which is described in detail elsewhere (Lisi et al., 2021a). A sub-sample of Brazilian mothers living in the Porto Metropolitan Area, Porto District and Braga District was selected from the baMBINO’s cohort. Mothers who agreed to be recontacted after taking part in the baMBINO’s survey were invited by telephone to participate in this study. In Brazil, participants were recruited at two private clinics (an obstetric/gynecologic clinic and a pediatric clinic) located in the municipality of Osasco in the Metropolitan Region of São Paulo. São Paulo was selected as a preferential site for research because the largest group of Brazilian immigrants interviewed in Portugal were born in that state. The participants recruited at the obstetric and gynecological clinic were informed about the study by the medical staff after their postnatal consultations, while participants recruited at the pediatric clinic were informed by trained administrative staff.

All participants received a Study Information Sheet and were given the opportunity to clarify any doubts before starting the interview. Written consent was obtained from all participants. Interviews were carried out by the first author, who is an Italian medical doctor fluent in Portuguese, and who was a full-time PhD student in her early 30s and had no children when the data were collected. The interview guide explored a variety of topics related to infant feeding practices, the influence of HMS marketing and, in the case of immigrant Brazilians, also migratory experience.

Sociodemographic and other infant feeding related data were also collected. While the interviewer’s age might have lessened the gap between her and younger participants, her lack of experience as a mother might have positioned her as an outsider. However, this may also have contributed to her adopting a more neutral attitude towards participants’ infant feeding practices, by not identifying herself with any preferences. The interview guide and the sociodemographic questionnaire are provided in Supplementary Materials.

Brazilian immigrants were interviewed at the date, time, and location most convenient for them (e.g., place of work, home, a coffee shop), while Brazilian natives were interviewed at the clinics following their consultations. These differences in data collection resulted from the opportunities that were available to the interviewer to recruit participants in the two countries where the study took place. We acknowledge that this might have influenced the relationship established between the interviewer and study participants, especially when the interviews occurred in healthcare settings. However, to minimize any arising power imbalances, the first author opted to (a) introduce herself as a researcher, and never as a medical doctor; and (b) position herself at a 45-degree angle from participants, when interviews took place at a healthcare setting. Furthermore, her own migratory experience to Portugal contributed to establishing empathy with Brazilian immigrants and toward being aware of migration-related issues. Trustworthiness was ensured through prolonged engagement in the field, use of a reflexive journal, and thought documentation as data analysis proceeded.

Interviews lasted approximately 30–60 min. They were digitally recorded, transcribed verbatim, and reviewed for accuracy by the first author. Participants’ anonymity was ensured by identifying them through an alphanumeric code in all documents and any electronic database and by guaranteeing the confidentiality of their statements.

### Data Analysis

Participants’ characteristics were aggregated as numbers and proportions. Sociodemographic data were retrieved from the form completed before each interview. Data about breastfeeding intention, practice, and HMS marketing exposure were obtained through a quantification of qualitative data (Sandelowski et al., 2009). We reported breastfeeding practices at the moment of the interview, if the child was aged less than 6 months. Otherwise, recalled breastfeeding practices at 6 months postpartum were reported. Exclusive breastfeeding was defined as feeding the child only human milk (WHO, 2008). If the child was given any food or drink, including HMS, we classified it as any breastfeeding. Proportions were compared using Pearson’s chi-square or Fisher’s exact test, as appropriate. The analysis was performed using SPSS (Version 28.0) and reported *p* value ≤ .05 were considered statistically significant.

Qualitative data were analyzed by the first author using content analysis. A pre-defined coding framework was defined following the topic domains of the interview guide and emerging themes were added to the framework as analysis progressed (Stemler, 2000). NVivo software (QSR International) was used to assign codes to relevant expressions and sentences, to identify new themes and sub-themes, and to examine patterns and differences in participants’ perspectives considering their migration status and socioeconomic characteristics, in a reflexive and iterative manner. Additional details about the content analysis were provided in Table 1. The direct quotes from participants used to illustrate our findings were translated from Portuguese to English by the first author. They are presented throughout the text and additional quotes may be found in Supplementary Tables 1 and 2.

**Table 1.** Data Analysis Structure.

Theme	Definition	Sub-Theme	Definition
Breastfeeding beliefs and intentions	Participants’ intention to breastfeed their infant and influencing factors	Breastfeeding as the natural infant feeding choice	Breastfeeding as the natural way of feeding one’s own infant
		Memories about breastfeeding	Women recalling family members breastfeeding
		Breastfeeding as a dream	Desire of becoming a mother and breastfeeding one’s own infant
		Breastfeeding as the best choice	Breastfeeding as the best option to feed one’s own infant
		Emotional bond between mother and child	Mother-child attachment through the act of breastfeeding
		Previous breastfeeding experience	Multiparous women opting to breastfeed due to their previous breastfeeding experience
		Breastfeeding information	Primiparous women’s need to seek for breastfeeding information
Infant feeding practices and experiences	Type of infant feeding and participants’ experience with feeding their infants	Low human milk supply	Women’s perception of inadequate human milk production
		Return to work	Introduction of formula due to the need to return to work
		Formula brand recommended by health professionals	Women seeking for medical advice on the best formula brand in the market
		Convenience	Introduction of formula to better conciliate motherhood responsibilities with other responsibilities in the host country
		Infant refusal of the breast	Infant showing signs that he/she does not want to nurse
		Medical advice to introduce formula and food	Introduction of formula or food as a result of medical recommendation
		Awareness of difficulties associated with breastfeeding	Anecdotal reports of other mothers about the difficulties encountered in breastfeeding
		Idealized depictions of breastfeeding	Expectation of a positive breastfeeding experience contrasting with women’s lived experience
		Enjoyable experience	Breastfeeding described as a lovely experience despite difficulties and pain
		Feelings of sadness for not being able to breastfeed	Feelings of sadness associated with the impossibility of breastfeeding as initially planned
Exposure to human-milk substitutes marketing	Participants’ recall of being exposed to human-milk substitutes marketing	Paying attention to formula advertising	Women paying attention to formula advertising only when they need to buy them
Mothers’ perspectives on human-milk substitutes marketing	Participants’ feelings and opinions about human-milk substitutes marketing	Free formula samples as an incentive	Free formula samples are believed to induce formula use, thus discouraging breastfeeding
		Positive attitude because of formula price	Women who agree with free formula samples distribution in order to be able to try the product before buying an entire can of formula, which is expensive
		Positive attitude towards provision of free formula samples by a health professional	Favorable attitude towards free formula samples provision by a health professional when the provided formula is medically indicated
		Convenience	Formula advertising conveying the message that formula feeding is more convenient than breastfeeding
		Nutritional and health claims influence	Formula advertising conveying the message that infant formula is equivalent or superior to human milk
		Chubby formula-fed infant	Images of formula-fed chubby infants used in formula advertising are associated with healthy infants
		Appealing for mothers with breastfeeding problems	Formula advertising inducing formula feeding among mothers experiencing breastfeeding problems
		Product in-store placement	Product visibility and in-store placement inducing women to buy formula in an indirect way
		Expectation of future need of formula	Formula advertising anticipating the need of using the milk product in the future
		Formula feeding norm in host country	Formula feeding perceived as more accepted by society in the host country

## Results

### Characteristics of the Sample

Immigrant and native Brazilians had a relatively similar level of education and household income. Most immigrants had been living in Portugal for more than 5 years and the majority were mothers to infants aged 6 months or older. Conversely, most natives were mothers to infants aged less than 6 months. These differences between the two groups were statistically significant. Immigrant participants were more exposed to free formula samples and HMS advertising, but these differences were statistically significant only for exposure to free formula samples. The number of native and immigrant Brazilians who breastfed exclusively or who opted for other types of infant feeding was similar (Table 2).

**Table 2.** A Comparison of Brazilian Native and Immigrant Participants’ Characteristics (*N* = 31).

Characteristic	Natives *n* = 16 (51.6) *n (%)*	Immigrants *n* = 15 (48.4) *n (%)*	χ^2^	*p*
Maternal age (years)				
18–24	2 (13)	1 (7)	4.316	.108
25–34	12 (75)	7 (47)		
≥ 35	2 (13)	7 (47)		
Infant age (months)				
< 6	13 (81)	3 (20)	11.630	<.001
≥ 6	3 (19)	12 (80)		
Maternal education				
Primary education/ none	0 (0)	2 (13)	3.505	.132
Secondary education	10 (63)	5 (33)		
Tertiary education	6 (38)	8 (53)		
Parity				
Multiparous	7 (44)	10 (67)	1.642	.200
Primiparous	9 (56)	5 (33)		
Marital status				
Married/Committed	15 (94)	15 (100)	.969	1.000
Single	1 (6)	0 (0)		
Length of stay in Portugal				
≤ 5 years	N/A	6 (40)	NA	NA
> 5 years	N/A	9 (60)		
Family household income				
Low	3 (19)	3 (20)	0.772	.877
Middle	9 (56)	10 (67)		
High	4 (25)	2 (13)		
Intention to breastfeed				
Yes	16 (100)	15 (100)	NA	NA
No	0 (0)	0 (0)		
Any breastfeeding				
Yes	14 (88)	12 (80)	.322	.654
No	2 (13)	3 (20)		
Exclusive breastfeeding				
Yes	9 (56)	9 (60)	.045	.833
No	7 (44)	6 (40)		
Exposure to free formula samples				
Yes	0 (0)	4 (27)	4.899	.043
No	16 (100)	11 (73)		
Exposure to HMS advertising				
Yes	9 (56)	13 (87)	3.476	.113
No	7 (44)	2 (13)		

*Note.* NA = not applicable; HMS = human milk substitutes; primary education = completed basic education; secondary education = completed high school; tertiary education = educational level achieved following the completion of high school. Family household income was calculated based on multiples of minimum wage (MW) as follows: ≤ 2 MW = low household income; > 2 MW and ≤ 6 MW = middle household income; > 6 MW = high household income. In Portugal, the MW was 580 Euro (= 684,98 US$) at the time of data collection. Since household income categories were pre-defined within the baMBINO project, we used 500 Euro (= 590,50 US$) as an approximation of MW. In Brazil, MW was 1163,55 Real (= 294,89 US$). Among Brazilian natives, two women planned to introduce formula before 6 months because of returning to work and one intended to introduce fruit at 4 months.

### Breastfeeding Beliefs and Intentions

Both natives and immigrant participants intended to breastfeed their infants. Most of them wished to continue breastfeeding for at least 1 year or for as long as possible. Breastfeeding was described as a “natural thing” and it was considered the best option by all participants (Supplementary Table 1). Some of them recalled how common it was for children to be breastfed in their families. Nevertheless, three native participants planned to introduce formula because they had to return to work.

Breastfeeding was also described as necessary to experience full motherhood and as something planned out since childhood, although this was more commonly expressed by native Brazilians: “I think if I didn’t breastfeed, I wouldn’t be a complete mother” (Native 4).

Both native and immigrant participants recognized the nutritional, immunological, and health benefits of human milk and highly valued the mother–child bond established through breastfeeding. Some natives believed that shorter breastfeeding duration prevented them from developing an emotional bond with their infants, who were reported by their mothers to be more susceptible to disease after breastfeeding discontinuation. Among multiparous immigrant participants, previous breastfeeding knowledge influenced the intention to continue breastfeeding, while primiparous participants expressed the need to seek information about breastfeeding more often.

### Infant Feeding Practices and Experiences

Despite their initial breastfeeding intentions, several participants encountered problems that led them to introduce formula. Among native Brazilians, two participants abandoned breastfeeding and switched to formula and five opted for mixed feeding (i.e., a combination of human milk and formula). The reasons given by participants to introduce formula were the baby crying, prolonged sucking and sleeping problems, and low infant weight, which was interpreted as being caused by low milk supply (Supplementary Table 1). In one instance, formula was introduced to enable the child to get used to it before the mother’s return to work.

Most native participants trusted health professionals to select the best infant formula brand in the market. Nevertheless, one low-income native participant opted for a formula type that she saw sponsored on television, without consulting with a pediatrician. She believed that the milk product advertised, which was cheaper than infant formulas, was adequate and of good quality: “The milk [formula] advertising is so good, that you believe it is very good. . . . It’s not just milk. They complement [it], you know, with proteins, sources of vitamins, you know?” (Native 3). However, the product was not appropriate for her infant’s age.

Several Brazilian immigrants also introduced formula in spite of their breastfeeding intentions. One participant switched to formula out of convenience. Another two discontinued breastfeeding after several unsuccessful attempts related to baby refusal of the breast and to perceived low milk supply. Two immigrant participants who were still breastfeeding stated that they introduced formula because of medical advice in the aftermath of infant weight concerns. One of them was even encouraged to stop breastfeeding, but she nevertheless persisted. Another participant was told by a health professional to introduce food before her child reached 6 months.

Participants’ expectations of breastfeeding sometimes contrasted with a less positive lived experience of feeding their infants human milk. While both native and immigrant primiparous participants were aware that breastfeeding can be a difficult practice, native Brazilians stated how public discourse in Brazil tends to address it in an idealized fashion, which is reinforced by breastfeeding campaigns. Nevertheless, although some natives and immigrants reported pain or difficulties in breastfeeding (caused by sore, flat, or inverted nipples, or mastitis), they considered it an overall positive and enjoyable experience that brought them feelings of sadness when they were not able to breastfeed as planned.

### Exposure to HMS Marketing

Brazilian immigrants were more exposed to HMS marketing than natives. For example, exposure to free formula samples was reported only by immigrants and it occurred in several settings: (a) during infant care workshops organized by private labs in the form of gift bags also containing other items (e.g., pacifiers); (b) in health care settings, and by a health professional after medical recommendation to use formula; and (c) by post, after registration at a baby club (although only the follow-on formula type). Although differences in HMS advertising were not significant, Brazilian immigrants were targeted by several formal marketing strategies. Almost all immigrant participants reported exposure to all forms of marketing at retail points (i.e., pharmacies and supermarkets), including discounts (e.g., “buy two, get one free”), catalogues, special displays, posters, and product visibility on the shelves. Conversely, among natives, only one participant was exposed to discounts and another two recalled product visibility at retail points. One native participant observed an increase of product diversification, although not in advertising: “I have the impression that the market has grown a lot, with more products, but without advertising. At the same time, there is a stimulus to give human milk” (Native 9).

Television commercials were also reported by immigrants and natives, as were magazine advertisements, although to a much smaller extent. Social media marketing, websites, and email promotions were indicated by some immigrant participants, and formula promotion through “mom Instagram influencers” and a WhatsApp video were reported by natives. In health care settings, industry-sponsored brochures were recalled by one native and two immigrant participants, whereas one immigrant participant was exposed to a poster of a formula company. However, some natives and almost half of immigrant participants stated that advertising generally did not get their attention, unless they needed to buy formula (Supplementary Table 2).

### Participants’ Perspectives on HMS Marketing

Compared to natives, immigrants more often expressed concern about how free formula samples’ distribution might discourage breastfeeding, namely by inducing mothers to test them on their child and then ending up switching to formula: “When they are small [babies], free samples. . . . I am against it because it is an incentive to not breastfeed” (Immigrant 15). Conversely, native participants manifested positive attitudes towards free formula samples more frequently than immigrants because, as they reasoned, infant formula is high priced, and that offered them an opportunity to try the product without having to buy an entire can. Only two participants were favorable to free samples provision by a health professional, provided that formula was medically indicated.

Both immigrants and natives believed that formula advertising might incentivize HMS introduction, namely by transmitting a message of convenience—especially to new mothers—or the idea that infant formula is nutritionally equivalent or superior to human milk. The latter was observed especially among mothers with low nutritional literacy. Immigrants warned that advertising induces mothers into believing that formula-fed chubby infants are healthy infants. Moreover, both immigrants and natives believed that formula advertising and visibility may be especially appealing for mothers experiencing breastfeeding difficulties. Product visibility and in-store placement were also perceived as a subliminal invitation to buy formula.

Some native participants stated that formula advertising made them think of what their child’s future feeding needs would be and even offered a feeling of relief and hope by enabling them to identify and eventually lean on an alternative to human milk, should the need ever arise. For an immigrant participant this anticipation of an infant’s feeding needs was associated with consumerism in the host country:

They [future mothers] think: “maybe it’s better to buy [a formula can] now [before birth]. I have to take advantage of it [discounts]. I can take a can or two already, so they’re already at my house [when the baby is born]. (Immigrant 9)

Some immigrants living in Portugal for over 5 years perceived formula feeding to be the norm in the host country. One even stated that she had been encouraged to discontinue breastfeeding and to switch to formula by her native Portuguese clients: “Here [they tell me] . . .: ‘I can’t believe you’re still breastfeeding this child’. . . . In the first months when as I working. . . I have a lot of clients, and they always said: ‘Buy milk [formula]’” (Immigrant 7). This contrasted with Brazilian immigrants’ perception of strong breastfeeding promotion in their home country through breastfeeding campaigns involving endorsement by female celebrities, social media, and social mobilization (e.g., Breastfeeding Week): “There are many programs on TV, on the radio, many advertisements and incentives [to breastfeed]. In soap operas that is very common” (Immigrant 16).

It is worth noting that, when asked about HMS advertising, some native participants actually recalled breastfeeding campaigns rather than industry marketing: “Normally. . .when I think about advertising, I think about the mother breastfeeding, not the mother giving the bottle” (Native 9).

## Discussion

Although Brazilians who migrated to Portugal reported having greater contact with HMS marketing in the host country, their breastfeeding intention and practices were similar to those of native Brazilians. This appears to be explained by a strong breastfeeding culture that had been passed on to them since childhood, invigorated and sustained through continuous policy about breastfeeding promotion in their country of origin. As previously reported by Lindsay et al. (2017), breastfeeding has been considered the infant feeding norm in Brazil. These perspectives were echoed by Brazilian natives in our study for whom the act of breastfeeding was strongly associated with motherhood. Brazilian immigrants living in Portugal also perceived breastfeeding as the best option to nurture their infants. Aside from recognizing the nutritional benefits of human milk for the child, both immigrants and natives believed that the act of breastfeeding was crucial in establishing an emotional bond with the child (Rocha et al., 2018). These cultural factors may help explain why a similar proportion of immigrant and native Brazilians were exclusively breastfeeding their infants, even though immigrants were more exposed to HMS marketing.

Breastfeeding intentions are predictive of better breastfeeding outcomes (Xu et al., 2021) and might have contributed to immigrant participants being less permeable to market factors exposure in the host country (Lisi et al., 2021b). Several participants compared HMS advertising messages to the information conveyed through breastfeeding promotion campaigns in their home country, distilling the former’s potential to induce unnecessary formula use and its negative consequences (e.g., premature breastfeeding cessation). This awareness appears to have been built through early exposure to strategies aimed at sensitizing both institutional and lay stakeholders to the importance of breastfeeding (Leal et al., 2018). These strategies have been more vigorously implemented since 1981, when the IC was adopted, through mass media and advertisements printed on lottery tickets, bills, and bank statements (Rea, 2003). Yet, prior to 1988, when the IC was first implemented, breastfeeding was already promoted through commercials featuring artists’ and celebrities’ testimonials. Together, these efforts contributed to increase social mobilization and women’s engagement in breastfeeding, which fostered a sociocultural context in Brazil highly supportive of breastfeeding (Rollins et al., 2016).

Brazilian immigrants were more aware of the potential negative influence of HMS marketing than natives. Their perspectives may have been influenced by contact with a host context upholding cultural beliefs at contrast with their own beliefs and where formula feeding is perceived as more socially accepted. Contrary to what has previously been reported by researchers, however, this clash between the perceived cultural values and infant feeding norms prevailing in the home and host countries did not result in formula being introduced (Joseph et al., 2018). Indeed, our findings did not point to a decline in breastfeeding intentions nor in exclusive breastfeeding in the host country. Nevertheless, Brazilian immigrants were subject to more industry marketing than before migrating, which may increase the risk of breastfeeding discontinuation under particular circumstances (Lisi et al., 2021b). For example, reduced or lack of family support linked to migration may lead immigrant women to buy formula out of need or for convenience. Furthermore, immigrants tend to rely more on health professionals in the host country (Joseph et al., 2018). Marketing through health professionals might be more powerful than direct-to-consumer strategies (e.g., formula samples sent by post or advertisements) in inducing formula use (Rothstein et al., 2020; Waite & Christakis, 2016). Health professionals are considered a reliable source of information about infant feeding. Thus, the receipt of a free formula sample by a health professional might have been interpreted as a product endorsement (Baker et al., 2020). Prescribing behaviors are also influenced by educational events, training, and research, which are often sponsored by the HMS industry (Baker et al., 2020). In our study, some immigrant participants reported feeling discouraged from exclusively breastfeeding following a consultation with a health professional, suggesting that immigrants might have been more affected by professional advice that has been influenced by industry marketing. Future research is needed to better understand how HMS marketing through health professionals may influence immigrants’ breastfeeding intention and practice.

Finally, both native and immigrant Brazilians were exposed to several marketing strategies. Native participants reported exposure to formula commercials on television and advertising by social media mom influencers in Brazil, pointing to possible violations of the IC despite existing strong national legislation (Law No. 11.265, 2006). In contrast, immigrant participants reported exposure to infant formula samples and other industry-sponsored products through participation in private-sponsored workshops and registration in baby clubs (Harris & Pomeranz, 2020; Hastings et al., 2020), a form of direct-to-consumer marketing forbidden by the IC but not by Portuguese legislation (Decree-Law 62, 2017). In one instance, an infant formula sample was provided by a health professional, contravening national regulations protecting exclusive breastfeeding from similar practices (Lisi et al., 2021b).

We believe that our study contributes to a deeper understanding of Brazilian immigrants’ perspectives about the influence of HMS marketing on breastfeeding outcomes. Although the analysis was conducted by the first author, thus preventing researcher triangulation, the quality of the findings was ensured through data sources triangulation (i.e., application of the same method to informants in two different settings). Further research should be done to investigate to what extent the results observed in our study might be related to immigration status rather than the country of residence, by comparing the perspectives of Brazilians living in Portugal with those of Portuguese natives. Our study also points to the importance of developing and implementing measures to reduce the influence of market factors and increase the dissemination of breastfeeding promotion strategies in Portugal.

### Limitations

Participants were defined as women based on the sex assigned at birth and were not asked about their gender identity. We acknowledge that this might have prevented an inclusive approach respectful of gender diversity. Unlike Brazilian immigrants who were recruited from a pool of participants giving birth at public maternities, native participants were recruited at private clinics, which might point to the latter having a higher socioeconomic status. However, some of the immigrant participants referred to having used or that they would have used the private health care sector if they were in Brazil, suggesting that this bias might be lower than expected.

Infants born to immigrant participants were older than those born to natives, thus increasing the risk of recall bias among immigrants. Additionally, exposure to marketing was self-reported and may have been subject to recall bias as well. Nevertheless, immigrants reported higher exposure when compared to natives. This might be due to the wide diversity of HMS marketing strategies to which they were subject in Portugal. Furthermore, there is a risk of participant bias, meaning that some participants’ answers might have been shaped by social desirability. We believe that the use of indirect questions asking whether pregnant women and mothers may have been influenced by HMS marketing might have contributed to more truthful answers.

Additionally, data were analyzed only by the first author, thus preventing a check of coding consistency and comprehensiveness, and introducing a potential for researcher bias. However, findings were reviewed with the co-authors and that might have reduced this bias. Finally, it should be noted that qualitative data analysis cannot demonstrate causal relationships, and that our findings aim to generate in-depth knowledge of participants’ contact and experiences with human milk substitutes in two settings with substantially different approaches to HMS marketing.

## Conclusions

Although Brazilian immigrants reported being subject to HMS marketing more often than Brazilian natives, the majority did not change their breastfeeding intention and practice after moving to Portugal. Brazilian immigrants were also more aware about the potential negative influence of HMS marketing on optimal breastfeeding outcomes. Sociocultural factors, namely strategies promoting breastfeeding and a strong breastfeeding culture in their home country, may have played an important protective role against the HMS marketing influence on breastfeeding intention and practice.

## Supplemental Material

sj-docx-1-jhl-10.1177_08903344221104717 – Supplemental material for The Influence of Human-Milk Substitutes Marketing on Breastfeeding Intention and Practice among Native and Immigrant Brazilians Click here for additional data file. Supplemental material, sj-docx-1-jhl-10.1177_08903344221104717 for The Influence of Human-Milk Substitutes Marketing on Breastfeeding Intention and Practice among Native and Immigrant Brazilians by Cosima Lisi, Henrique Barros, Alexandre Faisal-Cury, Alicia Matijasevich and Cláudia de Freitas in Journal of Human Lactation

sj-docx-2-jhl-10.1177_08903344221104717 – Supplemental material for The Influence of Human-Milk Substitutes Marketing on Breastfeeding Intention and Practice among Native and Immigrant Brazilians Click here for additional data file. Supplemental material, sj-docx-2-jhl-10.1177_08903344221104717 for The Influence of Human-Milk Substitutes Marketing on Breastfeeding Intention and Practice among Native and Immigrant Brazilians by Cosima Lisi, Henrique Barros, Alexandre Faisal-Cury, Alicia Matijasevich and Cláudia de Freitas in Journal of Human Lactation

sj-docx-3-jhl-10.1177_08903344221104717 – Supplemental material for The Influence of Human-Milk Substitutes Marketing on Breastfeeding Intention and Practice among Native and Immigrant Brazilians Click here for additional data file. Supplemental material, sj-docx-3-jhl-10.1177_08903344221104717 for The Influence of Human-Milk Substitutes Marketing on Breastfeeding Intention and Practice among Native and Immigrant Brazilians by Cosima Lisi, Henrique Barros, Alexandre Faisal-Cury, Alicia Matijasevich and Cláudia de Freitas in Journal of Human Lactation

sj-docx-4-jhl-10.1177_08903344221104717 – Supplemental material for The Influence of Human-Milk Substitutes Marketing on Breastfeeding Intention and Practice among Native and Immigrant Brazilians Click here for additional data file. Supplemental material, sj-docx-4-jhl-10.1177_08903344221104717 for The Influence of Human-Milk Substitutes Marketing on Breastfeeding Intention and Practice among Native and Immigrant Brazilians by Cosima Lisi, Henrique Barros, Alexandre Faisal-Cury, Alicia Matijasevich and Cláudia de Freitas in Journal of Human Lactation
