# Supplementary material for: The Influence of Human-Milk Substitutes Marketing on Breastfeeding Intention and Practice among Native and Immigrant Brazilians
Source: J Hum Lact. 2022 Jul 6;38(4):711–22. doi: 10.1177/08903344221104717 (PMC9597140; doi:10.1177/08903344221104717)
Supplement: sj-docx-1-jhl-10.1177_08903344221104717 – Supplemental material for The Influence of Human-Milk Substitutes Marketing on Breastfeeding Intention and Practice among Native and Immigrant Brazilians [file sj-docx-1-jhl-10.1177_08903344221104717.docx]

**Interview guide^[[1]](#footnote-1)^**

| Questions | Topics/Suggestions |
| --- | --- |
| I. Intentions and feeding practices | |
| 1. While you were pregnant of your most recent baby, did you plan how you would feed him/her? 2. After birth, was it possible to feed your baby according to what you planned? 3. (For mothers who breastfed) Overall, how was your experience with breastfeeding? 4. (For mothers who breastfed) For how long have you breastfed? Did you plan in advance how long would you like to breastfeed your baby? | [If yes] How did you plan to do it? What factors influenced your decision?  [If no] What were the reasons for changing the initial plan?  [If yes] Did you breastfeed for as long as you had planned? If not, why did you stop breastfeeding? |
| II. Expectations about the information provided and support to infant feeding practices | |
| 1. Have you ever had doubts about your baby's feeding? 2. In your opinion, what is the role that health professionals should play in regard to infant feeding? 3. Have you received any information about how to feed your baby from a health professional? 4. Did you receive any support to feed your baby from a health professional? | [If yes] Have you tried to clarify these doubts?  [If yes] In what way did you try to clarify them? By your own or have you asked for help?  [If yes] What kind of information did you receive? What did you think about the information provided?  [If no] Would you like to have received information from a health professional? What kind of information?  [If yes] What kind of support? What did you think about the support provided?  [If no] Would you have received support from a health professional? If yes, why? |
| III. Perspectives on the influence of industry marketing on infant feeding practices | |
| 1. Do you recall seeing artificial baby milk advertising (e.g., ads, leaflets)? If so, where (e.g., health center, hospital, pharmacy, in a shop, in the street)? 2. In your opinion, what impressions and feelings are aroused by the advertising of artificial baby milk in pregnant women and mothers? 3. Do you recall seeing free samples of artificial baby milk being distributed? If so, where (e.g., health center, hospital, pharmacy)? 4. What is your opinion about the distribution of free samples of artificial baby milk? 5. Do you recall seeing pacifiers being distributed for free? If so, where (e.g., health center, hospital, pharmacy)? 6. What is your opinion about the free distribution of pacifiers? | What do you remember about artificial baby milk advertising?  To what extent has advertising of artificial baby milk influenced your decisions about feeding your baby?  To what extent has the distribution of artificial baby milk free samples influenced your decisions about feeding your baby?  To what extent has the free distribution of pacifiers influenced your decisions about feeding your baby? |
| Perspectives on the context of the country of birth |  |
| 1. To what extent could your experiences with feeding your baby have been different if you were in Brazil? |  |
| Finalization | |
| 1. Is there anything you want to share with me that I have not asked about your baby's feeding experience? 2. Do you have any questions that you would like to ask me? |  |

1. This interview guide was originally written in English to integrate the study protocol that was submitted in English to the Ethics Committee. For the purposes of the interviews, it has been translated into Portuguese and it was administered in that language. [↑](#footnote-ref-1)
