# Supplementary material for: The Influence of Human-Milk Substitutes Marketing on Breastfeeding Intention and Practice among Native and Immigrant Brazilians
Source: J Hum Lact. 2022 Jul 6;38(4):711–22. doi: 10.1177/08903344221104717 (PMC9597140; doi:10.1177/08903344221104717)
Supplement: sj-docx-2-jhl-10.1177_08903344221104717 – Supplemental material for The Influence of Human-Milk Substitutes Marketing on Breastfeeding Intention and Practice among Native and Immigrant Brazilians [file sj-docx-2-jhl-10.1177_08903344221104717.docx]

**Sociodemographic questionnaire used with immigrant Brazilian women**

**Questionário sociodemográfico**

**Qual é a sua data de nascimento (**Dia / mês / ano**)? _____________**

**A Sra. é natural de onde?**

Estado **__________**

Cidade **__________**

**Qual é o grau de ensino mais elevado que completou?**

 Escola Primária (4º ano)

 Ensino Básico (9º ano)

 Escola Secundária (12º ano)

 Ensino Superior/Certificado ou Diploma pós-secundário (CET, CTESP, Bacharelato, Licenciatura)

 Formação superior avançada (Mestrado, Doutoramento)

 Nenhum

| **Qual é o seu estado civil?**   Casada   União de facto (companheiros não casados)   Viúva   Separada   Divorciada   Solteira  **Número** **de** **filhos:** _______ |  |
| --- | --- |
| **Pensando em todo o seu agregado familiar, em que escalão se encontra o seu rendimento bruto atual mensal (valor antes de serem retirados os impostos)?**   < 500€   500€ a 1000€   1001€ a 2000€   2001€ a 3000€   > 3000€  **Quantas pessoas vivem deste rendimento? (Incluindo o bebé) _____________** | |

**Há quanto tempo vive em Portugal?**

*(Quantidade TOTAL de tempo vivido neste país)*

_______(dias) _______ (semanas) ______ (meses) ______ (anos)

**De forma:**

Continua

Intermitente
