# Supplementary material for: The Influence of Human-Milk Substitutes Marketing on Breastfeeding Intention and Practice among Native and Immigrant Brazilians
Source: J Hum Lact. 2022 Jul 6;38(4):711–22. doi: 10.1177/08903344221104717 (PMC9597140; doi:10.1177/08903344221104717)
Supplement: sj-docx-3-jhl-10.1177_08903344221104717 – Supplemental material for The Influence of Human-Milk Substitutes Marketing on Breastfeeding Intention and Practice among Native and Immigrant Brazilians [file sj-docx-3-jhl-10.1177_08903344221104717.docx]

**Sociodemographic questionnaire used with native Brazilian women**

**Questionário sociodemográfico**

1. **Qual é a sua data de nascimento (**Dia / mês / ano**)? _____________**
2. **A Sra. é natural de onde?**

Estado **__________**

Cidade **__________**

1. **Até que série a Sra. completou na escola?**

**____________** série

**____________** grau

1. **A Sra. completou a faculdade?**

Sim

Não

1. **A Sra. completou:**

Mestrado  Sim  Não

Doutorado  Sim  Não

☐ N/A

1. **Em total, quantos anos completos de educação você completou?**

_______

1. **Qual é o seu estado civil?**

Casada

União de facto (companheiros não casados)

Viúva

Separada

Divorciada

Solteira

**7. Qual é a data do nascimento do seu bebé mais recente** (Dia / mês / ano)**?**

**_____________**

1. **Quantas gravidezes teve no total (incluindo esta)? _____**
2. **Quantas gravidezes terminaram com um aborto espontâneo? _______**

N/A

1. **Quantas gravidezes terminaram com aborto (Interrupção voluntária ou Interrupção médica) _______**

☐ N/A

1. **Quantas gravidezes terminaram com morte fetal/nados mortos (bebé morreu antes de nascer)? ________**

☐ N/A

1. **Quantos nascimentos resultaram em nados vivos, antes de completar as 37 semanas? _______**

☐ N/A

1. **Quantos nascimentos resultaram em nados vivos, depois de completar as 37 semanas? _______**

☐ N/A

1. **Quantas criançãs, suas filhas, vivem consigo (incluindo o bebé mais recente)?**

**_______**

1. **No mês passado, quanto receberam as pessoas da casa?**

(Não anotar centavos. 9------9= IGN)

**15.1 _____________________ R$ _________**

**15.2 _____________________ R$ _________**

**15.3 _____________________ R$ _________**

**15.4 _____________________ R$ _________**

1. **A família tem outras fontes de renda?**

**16.1 _____________________ R$ _________**

**16.2 _____________________** **R$ _________**

☐ Não tem outras fontes de renda

1. **Quantas pessoas vivem deste rendimento? (Incluindo o bebé)**

**____________**
