# Supplementary material for: The Influence of Human-Milk Substitutes Marketing on Breastfeeding Intention and Practice among Native and Immigrant Brazilians
Source: J Hum Lact. 2022 Jul 6;38(4):711–22. doi: 10.1177/08903344221104717 (PMC9597140; doi:10.1177/08903344221104717)
Supplement: sj-docx-4-jhl-10.1177_08903344221104717 – Supplemental material for The Influence of Human-Milk Substitutes Marketing on Breastfeeding Intention and Practice among Native and Immigrant Brazilians [file sj-docx-4-jhl-10.1177_08903344221104717.docx]

**Supplementary Table 1**

*Breastfeeding Beliefs and Intentions, and Infant Feeding Practices and Experiences – Supplementary Quotes.*

| Theme | Sub-theme | Supporting quotes | |
| --- | --- | --- | --- |
|  |  | Brazilian natives | Brazilian immigrants |
| 1. Breastfeeding beliefs and intentions | 1.1 Breastfeeding as the natural infant feeding choice | [1.1a] “I always counted on that [breastfeeding]. No, I didn't make plans.” (Native 6) | [1.1b] “Ah, I think it's...a natural thing.” (Immigrant 2) |
|  | 1.2 Memories about breastfeeding | [1.2a] “Everyone in my family has always breastfed.” (Native 2) | [1.2b] “My mother breastfed me until… a long time.” (Immigrant 11) |
|  | 1.3 Breastfeeding as a dream | [1.3a] “I think since I was little... since I dreamed of having a baby... I was going to breastfeed.” (Native 2) |  |
|  | 1.4 Breastfeeding as the best choice | [1.4a] “The issue of both food, immunity, affection, comfort, and everything. So that's why I wanted it.” (Native 6) | [1.4b] “What I really think is that breastmilk is better...” (Immigrant 13) |
|  | 1.5 Emotional bond between mother and child | [1.5a] “It's a bond between me and her... it's a special moment!” (Native 2) | [1.5b] “…my daughter loves breastmilk. It is a very enjoyable contact between mother and daughter, right?“ (Immigrant 8) |
|  | 1.6 Previous breastfeeding experience | [1.6a] “Because I couldn't breastfeed my [previous] son, my milk completely dried up due to the antibiotics I was taking. So I didn't have that bond with him, did I? (Native 17)  [1.6b] “It's because my other daughter... I took her off my breast at four months. Then she got really sick... That's why I wanted, with this one, to give her breastmilk.” (Native 11) | [1.6c] “I already breastfed the first one for many months and [with] the second one I wanted to do the same thing”. (Immigrant 7) |
|  | 1.7 Breastfeeding information | [1.7a] “So, actually, I did a lot of research, talking to the gynecologist, talking to friends, and research that I see on the Internet.” (Native 13) | [1.7b] “When I got pregnant, I researched a lot about everything, about childbirth, about… breastfeeding”. (Immigrant 10) |
| 1. Infant feeding practices and experiences | 1.1 Low milk supply | [1.1a] “Because the milk didn't do it... breastfeeding [was not enough] for him. He screamed of hunger.” Native 3  [1.1b] “So when I see that he is not satisfied with the last feeding of the night [and that] he is not able to sleep because of hunger, then I give him a little supplement.”( Native 9) | [1.1c] “I had milk. Then it started to come out [only] a little and I bought the pump. And then it came out at 40 ml, 60 ml. And then I stopped. Then I introduced powdered milk.” (Immigrant 10) |
|  | 1.2. Return to work | [1.2a] “When I'm not with him [due to work], then he will take another milk, other foods. And when I'm with him, [it will be] breastmilk, up to 2 years… He's starting to get formula to get used [to it]”. (Native 7) |  |
|  | 1.3. Formula brand recommended by health professionals | [1.3a] “I just asked if I could use this milk [formula] that I already had in mind. Then he [health professional] said that it is one of the best milks. And that everything else [provided by that formula] is very good, regarding the nutrients that are essential at this stage.” (Native 1)  [1.3b] “I was previously advised by the pediatrician and he had already prescribed artificial milk [at the hospital, after birth]. I just had not used it yet [after discharge]. I kept insisting on breastmilk. But then it wasn't enough for her.” (Native 5) |  |
|  | 1.4 Convenience |  | [1.4a] “It was really my choice. Just to... to make my life easier.” (Immigrant 16) |
|  | 1.5 Infant refusal of the breast |  | [1.5a] “The baby has to suck to be able to have milk and, in this case, he didn't want any more and so I continued with the bottle.” (Immigrant 10) |
|  | 1.6 Medical advice to introduce formula and food |  | [1.6a] “In the consultations to weigh her, she [family nurse] saw that she was losing a lot of weight, so she introduced the supplement.” (Immigrant 12)  [1.6b]: “In the first few days, when the nurse came to my house to make the heel prick screening, she saw that she [the baby] was not gaining the weight that was necessary, so she recommended the introduction of artificial milk. And at the time, she even advised me to stop breastfeeding and to just stick to artificial milk. And I said that I would like to continue breastfeeding.” (Immigrant 13) |
|  |  |  | [1.6c] “When he reached the fourth month, four and a half months, the pediatrician saw that he was gaining [weight] and such, but that he needed to feed from other sources. So she told us to introduce the porridge and to introduce the soup.” (Immigrant 6) |
|  | 1.7 Awareness of difficulties associated with breastfeeding | [1.7a] “Because before having breastfed, [before] having this experience, we listen to a lot of stories and we hear stories: ‘Oh, because it hurts… because it bleeds…’” (Immigrant 15) | [1.7b] “Then you know, everyone says: ‘Right, that it's all very bad.’ ‘Oh, the nipple cracks!’ ‘Oh, it hurts a lot!’ I had no problem.” (Immigrant 8) |
|  | 1.8 Idealized depictions of breastfeeding | [1.8a] “Breastfeeding is not that beautiful thing that everyone says it is. It hurts like hell.” (Native 9)  [1.8b] “Here, in Brazil there is a campaign on breastfeeding. The campaign is very strong. So you see a lot of actresses [saying]: ‘Breastfeed your child [until] he's two years old’. But they don't talk about the painful aspects. So, when you live it in reality, you find it's not like that at all.” (Native 3) |  |
|  | 1.9 Enjoyable experience | [1.9a] “In the beginning, in the first fifteen days, it was very painful, complicated... but it is very pleasant to feed, to breastfeed.” (Native 1) | [1.9b] “And that is very enjoyable. It's a very enjoyable act.” (Immigrant 8) |
|  | 1.10 Feelings of sadness for not being able to breastfeed | [1.10a] “But I feel sorry for not being able to breastfeed.” (Native 1) |  |
